# Does antipsychotic therapy prevent the development of chronic psychotic disorders in people addicted to ilegal psychoactive substances

**DOI:** 10.1192/j.eurpsy.2023.1412

**Published:** 2023-07-19

**Authors:** S. S. Kasper, A. Čustović

**Affiliations:** 1 Hospital Department, Cantonal Institute for ADDiction, Zenica, Bosnia and Herzegovina

## Abstract

**Introduction:**

Acute psychotic disorders are increasingly being diagnosed in people addicted to PASA part of these patients develops chronic psychotic disorders for reasons that still insufficently known.

**Objectives:**

The aim of the study was to determine preventive potential of antipsychotics in the development of chronic psychotic disorders as well as possible side effects of theur use.

**Methods:**

The prospective retrospective qualitative study conducted in the period Septmeber 2017-September 2022.Data from medical records and electronic databases were used in the study.A structured questionnare for conductin research,a clinnical psychatic inteview,MMPI 202,tests to determine of ilegal PAS in body flluids.

**Results:**

According to the results of the study adequate treatment of the underlying desease,fewer or complete abscence of relapses,social and psychoteraapeutic support had the greater effects.In the group of opiate addicts an adequate dose of supstitution therapy it often played a crucial role.

**Conclusions:**

In experimental conditions the hypotesis about the preventive effect of antipsychotics on the development of psychotic disorders in peoplle addicted to PAS.On the contrary a whole series of new questions has beenopened.

**Disclosure of Interest:**

None Declared

